# Cross-Sectional Associations of Physical Fitness Performance Level and Sleep Duration among Older Adults: Results from the National Physical Fitness Survey in Taiwan

**DOI:** 10.3390/ijerph17020388

**Published:** 2020-01-07

**Authors:** Po-Fu Lee, Chien-Chang Ho, Ding-Peng Yeh, Chang-Tsen Hung, Yun-Chi Chang, Chia-Chen Liu, Ching-Yu Tseng, Xin-Yu Hsieh

**Affiliations:** 1 Graduate Institute of Sport Coaching Science, Chinese Culture University, Taipei City 111, Taiwan; f520184fred@yahoo.com.tw; 2 Department of Physical Education, Fu Jen Catholic University, New Taipei City 242, Taiwan; 0024@mail.sa.gov.tw (D.-P.Y.); james76630@hotmail.com (Y.-C.C.); 015844@mail.fju.edu.tw (C.-Y.T.); r173690@gmail.com (X.-Y.H.); 3 Research and Development Center for Physical Education, Health and Information Technology, College of Education, Fu Jen Catholic University, New Taipei City 242, Taiwan; 4 College of Humanities and Social Sciences, Taipei Medical University, Taipei City 110, Taiwan; 5 Department of Health and Leisure Management, Yuanpei University of Medical Technology, Hsinchu City 300, Taiwan; hongct@mail.ypu.edu.tw; 6 Department of Physical Therapy and Assistive Technology, National Yang-Ming University, Taipei City 112, Taiwan; 7 Department of Physical Education, National Taichung University of Education, Taichung City 403, Taiwan; jamesliu@gm.ntcu.edu.tw

**Keywords:** physical fitness, sleep duration, elderly, Taiwan

## Abstract

Research on relationships between physical fitness and sleep duration among older adults is scarce, especially in Taiwanese representative samples of elderly people who undergo physical fitness measurements. This study aimed to determine the associations between physical fitness and short and long sleep durations among older adults in Taiwan. We conducted a cross-sectional study and reviewed data derived from the National Physical Fitness Survey in Taiwan. A total of 24,125 Taiwanese adults aged 65 years and older participated in this study between October 2014 and March 2015. Each individual’s sleep duration was recorded with a standard questionnaire method. Sleep duration data were stratified into short (≤5 h), normal (6–7 h), and long (≥8 h) sleep duration groups. Physical fitness was assessed by five components: aerobic endurance (2 min step test), muscle strength and endurance (30 s arm curl and 30 s chair stand tests), flexibility (back scratch and chair sit-and-reach tests), body composition (body mass index (BMI) and waist-to-hip ratio (WHR)), and balance (one-leg stance with eye open and 8-foot up-and-go tests). To understand whether a dose–response relationship exists between physical fitness and short or long sleep duration, we analyzed four levels of performance on the basis of quartiles of physical fitness measurements by using logistic regression. The first quartile of physical fitness performance was the baseline level. The odds ratio (OR) for short sleep duration for the third quartile of BMI was 0.8031 times (95% CI, 0.7119–0.9061) lower than the baseline. For the fourth quartile of BMI, the OR was 0.8660 times (95% CI, 0.7653–0.9800) lower than the baseline. The adjusted OR for long sleep duration significantly decreased in the second, third, and fourth quartiles of the 30 s chair stand, back scratch, chair sit-and-reach test, one-leg stance with one eye open, and BMI. The adjusted OR was increased in the third and fourth quartiles of the 8-foot up-and-go and WHR. The results of the current study suggest that physical fitness performance may influence sleep duration as an associated factor, and the relationship is much stronger for long sleep duration than for short sleep duration.

## 1. Introduction

Research has indicated associations between sleep duration and cardiovascular disease development and its morbidity (such as myocardial infarction, angina, and stroke) [1], obesity [2], increased risk of metabolic diseases, and mental disorders [3,4]. Furthermore, sleep duration is also related to elderly peoples’ fitness and capabilities [1]. Specifically, Fex et al. [5] demonstrated that older subjects who reported getting over 9 h of sleep exhibited significantly lower performance levels on the chair stand, balancing with opened eyes, and muscle strength tests. Moreover, Stenholm and colleagues [6] stated that long sleep duration is associated with physical function decline relative to adults with normal (7–8 h) or short (<6 h) sleep duration. The evidence has generally indicated adverse effects from improper sleep duration on the older population. These age-related issues are essential for global socioeconomic development, especially for Taiwan, since it has been reported as an aged society [7]. Thus, how to achieve successful aging (SA) has become an important topic for modern society.

Although several factors on SA have been reported, sleep and physical fitness are crucial for SA. Specifically, research has indicated the positive association between sleep and SA among elders [8]. Besides, good sleep may increase a variety of domains in elderly people’s body functioning [9]. The connections among aging, sleep, and functional capacities among the elderly should be emphasized [8,10] to avoid disease and disability, as well as maintain their social connection [11,12]. Some studies have focused on the effects of standardized physical fitness test performance levels [7,13]. In particular, Lin et al. [7] investigated older Taiwanese adults and determined that physical fitness tests, including cardiopulmonary endurance, mobility, muscle strength, and balance, are statistically significant and crucial for SA. Moreno-Vecino et al. [10] indicated that sleep disturbance is associated with lower physical fitness in older adults. Studies have indicated the importance of physical fitness and sleep for older adults. However, although both physical fitness and sleep are vital, the relationship between these fundamental needs and how their interaction affects physical functions has seldom been discussed in the literature.

Improper sleep conditions are associated with reduced muscle mass and muscle function in older adults [14]. Fu et al. [15] suggested that muscle strength, balance, and walking speed are associated with long sleep duration in the elderly population of China. Functional capacity is influenced by sleep duration, health condition, and ethnicity [16,17]. However, because of limited knowledge, the link between physical fitness and sleep duration is still poorly understood. Therefore, the purpose of this study was to determine the associations between physical fitness performance and short and long sleep durations among older adults in Taiwan.

## 2. Material and Methods

### 2.1. Study Design and Participants

We conducted a cross-sectional study to determine the associations between short and long sleep durations and physical fitness measurements among Taiwanese older adults aged 65 years or over in the present study. Data were obtained from the 2014 and 2015 National Physical Fitness Survey in Taiwan (HPFST 2014–2015) database collected by Taiwan’s Sports Administration, Ministry of Education. The participants were recruited through 46 test stations among 20 cities in Taiwan. The test-related information, such as date, time, and location, were pre-announced to the local communities by health-care facilities, elder-specific classes or clubs, civil sports centers, and social media. The station usually accompanies events such as enterprise family day, national sports day, or elderly-related sports events, or could be in an exhibition or outside a concert hall. On the testing days, convenience sampling was applied for the stations. The recruiters were searching and invited elderly people to join the test. All participants needed to provide their identification document before they filled out the demographic characteristics and life-habit questionnaires. After that, they had to submit the measurement of resting heart rate and blood pressure for safe preliminary screening before conducting physical fitness measurements. The exclusion criteria for the recruitment and enrollment of participants were as follows: (1) resting heart rate ≥100 beats/min; (2) systolic blood pressure ≥ 140 mmHg or diastolic blood pressure ≥90 mmHg; and (3) having cardiovascular disease, hypertension, chest pain, vertigo, or musculoskeletal disease. Finally, we gave the questionnaire to participants and gathered physical fitness data from a total of 24,125 older adults (95.5% response rate); data were collected between October 2014 and March 2015. Written informed consent forms were obtained from all participants. This study was approved by the Ethical Committee of Fu Jen Catholic University in Taiwan, and informed consent was obtained from each participant.

### 2.2. Data Collection

A face-to-face interview and physical examination were completed by a well-trained research assistant and nurses. The data from the questionnaire included demographic characteristics (i.e., age, sex, education, and monthly income), life habits (i.e., smoking, betel nut chewing (seed of areca palm, grows in the tropical Pacific and parts of east Africa, causes increased risk of diseases, and possibly impairs physical fitness [18]), and dieting), and health status. All data were recorded. Anthropometric measurements, including body weight, height, waist circumference (WC), and hip circumference (HC), were carried out after the participants had removed their shoes and heavy clothes. Body weight was measured to the nearest 0.1 kg, using a weighing scale. Body height was measured to the nearest 0.1 cm, using a wall-glued metal measuring tape and an acute-angled headpiece while the subjects stood against a plumb-checked vertical wall and wore no shoes. The body mass index (BMI) was calculated as weight in kilograms divided by the square of the height in meters. The WC measurement (measured to the nearest 0.1 cm) was determined with the use of a soft measuring tape at the level of the natural waist, which was identified as the level at the hollow molding of the trunk when the trunk was bent laterally. The HC measurement (measured to the nearest 0.1 cm) was determined at the level of the greater trochanter. The waist-to-hip ratio (WHR) was also calculated. The cut-off BMI points for obesity status were adopted, as suggested by the Taiwan’s Ministry of Health and Welfare (MOHW), including underweight (BMI < 18.5 kg/m^2^), normal (18.5 ≤ BMI < 24 kg/m^2^), overweight (24 ≤ BMI < 27 kg/m^2^), and obese (BMI ≥ 27 kg/m^2^) categories [19].

### 2.3. Sleep Duration Assessment

Sleep duration was self-reported. The questionnaire investigated sleep duration with the following question: “How many hours do you usually sleep per day?” Each individual’s sleep duration was categorized into the short (≤5 h), normal (6–7 h), or long (≥8 h) sleep duration group. However, based on the characteristic of Taiwanese elderly population on continuous working [20], our analyses were performed by limiting sleep data to that collected on weekdays, to avoid the potential issue of weighted average sleep [21], such as recovery sleep on the weekend [22].

### 2.4. Physical Fitness Measurements

In order to figure out the functional capacity for older adults, we assessed five main components of physical fitness, which consisted of nine measurements: aerobic endurance (2 min step), muscle strength and endurance (30 s arm curl and 30 s chair stand), flexibility (back scratch and chair sit-and-reach), balance (one-leg stance with eye open and 8-foot up-and-go), and body composition (BMI and WHR). Each measurement of physical fitness had accompanying performance standards for men and women aged 65 years or over. These standards were based on an annual national survey of more than 20,000 Taiwanese people. Performance was measured by an examiner who had attended an official training seminar and had passed a certification test on standardized procedures. The 2 min step, 30 s arm curl, 30 s chair stand, back scratch, chair sit-and-reach, and 8-foot up-and-go were strictly performed according to the Serious Fitness Test (SFT) manual [23]; the one-leg stance with one eye open has been described in other articles [24].

The content of the measures was explained to the participants, and they had 10 min to prepare with a warm-up so that their best performance could be achieved. Measures were scheduled before any other exercise. All participants submitted to measurement in the following order, with sufficient rest (3–5 min) between measurements: body weight, height, WC, HC, one-leg stance with one eye open, 30 s chair stand, 30 s arm curl, 2 min step, chair sit-and-reach, back scratch, and 8-foot up-and-go. For each measurement, the participants were classified into one of four quartiles according to their physical fitness performance levels.

### 2.5. Statistical Analyses

Data were analyzed by using Statistical Analysis System (SAS) software package (Version 9.4, SAS Institute Inc., Cary, NC, USA). Differences in the subjects’ demographic data and the physical fitness measurements between categories of sleep duration were analyzed by one-way analysis of variance (ANOVA) or chi-square test. When a significant *F* value was found (*p* < 0.05), Tukey’s post hoc test was performed to determine the differences between the pairs of means. Logistic regression models were used to estimate the odds ratios (ORs) and 95% confidence interval (CI) for the association between quartiles of functional physical fitness measurements and short or long sleep duration, while adjusting for potential confounding variables (i.e., age, gender, education, monthly income, marital status, self-reported health status, smoking status, and chewing betel nuts). All data are expressed as means ± standard deviation (SD) or frequency percentage. The significance level adopted to reject the null hypothesis was *p* < 0.05.

## 3. Results

A total of 24,125 respondents were involved in the present study, consisting of 37% males and 63% females. The majority of the participants were not highly educated and had a high prevalence of obesity. Over 80% of participants lived with a disposable income below $20,000 NTD per month. For the self-reported health status, around 50–60% of respondents were “good”, “excellent”, or “very good” in health. Smoking and chewing of betel nut in the population were limited.

Table 1 presents the demographic characteristics of the different sleep duration groups. The respondents’ distribution among the groups is 17%, 51%, and 32% for short, normal, and long sleep duration, respectively. The results present no significant group differences in terms of obesity status, education, monthly income, marital status, self-reported health, smoking, and betel nut chewing. However, the long-sleep-duration group contained higher age, height, and body weight than other groups.

Table 2 lists the physical fitness measurements and group comparison of the research population. Besides BMI, almost all of the measurements performed show significant group differences in our results. Further, the results indicate that the normal-sleep-duration population had a better fitness performance, the short-sleep-duration group was second, followed by the long-sleep-duration group. Although we did not find a significant difference in BMI, the result indicated that long sleep duration was possibly related to being overweight and obese in terms of WHR.

Table 3 presents the result of the logistic regression analysis for short sleep duration. The fitness measurements were divided into quartiles. Then, the worst-performing quartile of each measurement was assigned as the reference. The other quartiles were used as the independent variables, to predict their odds on short sleep duration (dependent variable; dummy variable was applied). Thus, logistic regression models were conducted for the odds ratio (OR) of the quartiles within each measurement. Moreover, the adjusted models were conducted to eliminate the effects of individual differences. Potential influences such as age, gender, education, monthly income, marital status, self-reported health status, smoking status, and chewing betel nuts were used for confounding-factor adjustment. According to the results of Table 3, a statistically significant difference was found only on BMI after the adjustments. The third quartile of BMI had an OR value of 0.8031 (95% CI, 0.7119–0.9061), and the fourth quartile gave an OR value of 0.8660 (95% CI, 0.7653–0.9800). This implies that elders who have a lower BMI may have less opportunity to sleep for a short time (compare to highest BMI population).

Table 4 presents another logistic regression analysis for long sleep duration. Statistical significance was found for the 30 s chair stand, back scratch, chair sit-and-reach test, 8-foot up-and-go, one-leg stance with one eye open, BMI, and WHR. Specifically, elders who performed better with lower body muscle strength, flexibility, dynamic mobility, balance, and body composition presented around 13–27% lower odds of sleeping for a long time (compared to the worst performer’s group). However, the effects of cardiopulmonary fitness and upper body muscle strength on sleep duration remain unclear.

## 4. Discussion

The purpose of the present study was to determine the relationship between sleep duration and physical fitness in Taiwanese adults aged at least 65 years. A representative database was mainly used for the multivariate logistic regression analysis, based on the quartiles of each physical fitness measurement for different sleep durations. In our results, both short-term and long-term sleep were associated with physical fitness among older adults, and individuals with long-duration sleep (≥8 h/day) had the strongest association. In addition, both short-duration sleep (≤5 h/day) and long-duration sleep (≥8 h/day) were associated with older adult BMI. This finding is consistent with a previous study that identified an association between sleep duration and BMI [18]. This reveals that older adults, especially women, who sleep less than 5 h or more than 8 h a day may have an increased risk of obesity [25]. The association between sleep duration and BMI among physically fit older adults is clear.

Furthermore, long-duration sleep was associated with lower body endurance (30 s chair stand), upper body flexibility (back scratch), lower body flexibility (chair sit-and-reach test), dynamic balance (8-foot up-and-go), static balance (one-leg stance with one eye open), and WHR. Long sleep duration might be associated with some physical fitness performance in older adults. Generally, older adults who performed well on these tests were more likely than average to sleep longer than 8 h a day. Studies have stated that fit individuals have longer sleep duration, shorter sleep onset latencies [26,27], and higher levels of slow-wave sleep compared to unfit subjects [27,28]. Bocalini et al. [29] also found that older adults with relatively high physical fitness have a better quality of life, including their sleep. Our result seems to support this finding.

By contrast, short-duration sleep had only one significant association, namely, its association with BMI status. Nevertheless, the results indicated that, relative to the capabilities of unfit subjects, functional capabilities such as back scratch, 8-foot up-and-go, one-leg stance with one eye open, and WHR could be associated with short-duration sleep before the confounding-factor adjustment. The individual difference could be considerable in terms of the relationship between short-duration sleep and physical fitness. However, studies have indicated that sleep loss may reduce muscle function and total work capability [30,31], cause energy imbalance [32], and decrease aerobic and anaerobic power production [33,34]. The insignificant *p*-values of various results of the present study were unexpected. Future studies should focus on the effects of short-duration sleep on physical fitness.

The strength of the present study was the use of representative data for analysis. However, some limitations should be addressed. First, the research population was composed of Taiwanese adults aged 65 and older. Additional investigations should be conducted on subjects from different countries, of different races, and of different cultures. Second, the present study adopted a cross-sectional study design. No cause-and-effect relationship can be guaranteed. Future studies should adopt a longitudinal study design or use qualitative methods to understand the cause-and-effect relationships between sleep duration and physical fitness. Third, it is suggested that sleep quality is influenced by age, gender, health status, and the lack of information on insomnia, depression, stress levels, and other relevant phenomena [35,36,37]. Although the present study controlled for these confounding factors during the analysis, we suggest future studies focus on a more specific population in terms of the relationship between sleep duration and physical fitness in older adults.

In conclusion, this study suggests that physical fitness performance influences the sleep duration of older adults. Older adults who are fit tend to have a longer sleep duration than unfit older adults. This study demonstrated some limited evidence indicating that among Taiwanese older adults’ physical fitness is associated with short-duration sleep, although this does not include BMI status. However, further investigation is still needed.

## Figures and Tables

**Table 1:** Demographic characteristics of study subjects according to sleep-duration levels.

Variables	Sleep Duration (Hours)			*p*	Tukey’s Post Hoc Test
	Short (≤5 h)	Normal (6 to 7 h)	Long (≥8 h)		
No. of subjects	4035	12,393	7697		
Age (y)	73.48 ± 6.18	72.63 ± 6.03	74.04 ± 6.78	<0.0001 ^a^	L > S > N
Gender (% of men)	28.43%	36.08%	41.94%	<0.0001 ^b^	
Height (cm)	155.44 ± 7.80	156.85 ± 8.04	157.15 ± 8.21	<0.0001 ^a^	L > N > S
Body weight (kg)	59.97 ± 10.34	61.25 ± 10.22	61.54 ± 10.52	<0.0001 ^a^	L, N > S
Obese Status				0.0246	
Underweight	2.82%	2.15%	2.35%		
Normal weight	41.17%	39.75%	40.00%		
Overweight	30.91%	33.57%	32.77%		
Obese	25.10%	24.53%	24.88%		
Education				<0.0001 ^b^	
Elementary school or lower	65.00%	52.94%	57.73%		
Junior or senior school	25.19%	31.29%	27.56%		
College or higher	9.81%	15.77%	14.71%		
Monthly Income				<0.0001 ^b^	
≤20,000 NTD	89.41%	84.10%	86.58%		
20,001–40,000 NTD	6.78%	8.91%	7.88%		
≥40,001 NTD	3.81%	6.99%	5.54%		
Marital Status				<0.0001 ^b^	
Never married	48.70%	53.59%	53.74%		
Married	26.42%	27.35%	26.60%		
Divorced/widowed/other	24.89%	19.06%	19.66%		
Self-Reported Health Status				<0.0001 ^b^	
Excellent or very good	16.58%	20.92%	24.27%		
Good	38.12%	46.39%	43.37%		
Fair or poor	45.30%	32.69%	32.36%		
Smoking Status				<0.0001 ^b^	
Never	92.63%	91.71%	89.46%		
Current	4.62%	4.92%	6.23%		
Former	2.75%	3.38%	4.31%		
Chewing betel nuts				0.2180	
Never	96.97%	97.46%	97.09%		
Current	1.62%	1.23%	1.34%		
Former	1.42%	1.30%	1.57%		
Sleep duration (h)	4.54 ± 0.82	6.51 ± 0.5	8.41 ± 0.79	<0.0001 ^b^	L > N > S

Abbreviations: ANOVA, analysis of variance; S, short; L, long; N, normal; values are expressed as means ± standard deviation (SD) and %.; ^a^ one-way ANOVA, *p* < 0.05; ^b^ Chi-square test, *p* < 0.05.

**Table 2:** The comparison of physical fitness measurements according to sleep-duration levels.

Variables	Sleep Duration (Hours)			*p*	Tukey’s Post Hoc Test
	Short (≤5 h)	Normal (6 to 7 h)	Long (≥8 h)		
Men					
2 min step test	84.97 ± 30.25	87.73 ± 29.24	82.41 ± 30.59	<0.0001 ^a^	N > S > L
30 s arm curl	17.80 ± 6.29	18.31 ± 6.44	17.35 ± 6.32	<0.0001 ^a^	N > S, L
30 s chair stand	15.24 ± 5.51	15.82 ± 5.42	14.54 ± 5.53	< 0.0001 ^a^	N > S > L
Back scratch	−14.79 ± 13.70	−13.25 ± 13.23	−15.16 ± 13.83	<0.0001 ^a^	N > S, L
Chair sit-and-reach test	−0.80 ± 11.20	0.19 ± 10.89	−1.77 ± 10.99	<0.0001 ^a^	N > S > L
8-foot up-and-go	8.18 ± 3.20	7.71 ± 2.78	8.63 ± 3.54	<0.0001 ^a^	L > S > N
One-leg stance with eye open	13.01 ± 10.52	14.21 ± 10.57	11.84 ± 10.24	<0.0001 ^a^	N > S > L
BMI (kg/m^2^)	24.70 ± 3.34	24.83 ± 3.22	24.71 ± 3.16	0.215	
WHR	0.92 ± 0.08	0.92 ± 0.07	0.93 ± 0.07	0.026	L > N
Women					
2 min step test	82.43 ± 30.44	84.43 ± 29.17	79.84 ± 31.43	<0.0001^a^	N > S > L
30 s arm curl	17.19 ± 6.07	17.61 ± 6.21	16.83 ± 6.03	<0.0001 ^a^	N > S > L
30 s chair stand	14.33 ± 5.04	15.05 ± 5.08	14.03 ± 5.16	<0.0001 ^a^	N > S > L
Back scratch	−7.63 ± 12.02	−6.92 ± 11.53	−8.63 ± 12.09	<0.0001 ^a^	N > S > L
Chair sit-and-reach test	3.90 ± 10.12	4.39 ± 9.83	2.87 ± 9.70	<0.0001 ^a^	N, S > L
8-foot up-and-go	8.43 ± 2.97	8.08 ± 2.87	8.82 ± 3.40	<0.0001 ^a^	L > S > N
One-leg stance with eye open	11.71 ± 9.81	12.67 ± 10.19	10.72 ± 9.75	<0.0001 ^a^	N > S > L
BMI (kg/m^2^)	24.74 ± 3.60	24.80 ± 3.52	24.88 ± 3.62	0.271	
WHR	0.88 ± 0.08	0.88 ± 0.08	0.89 ± 0.08	<0.0001 ^a^	L > N, S
Total					
2 min step test	83.15 ± 30.40	85.62 ± 29.24	80.92 ± 31.10	<0.0001 ^a^	N > S > L
30 s arm curl	17.36 ± 6.14	17.86 ± 6.30	17.05 ± 6.16	<0.0001 ^a^	N > S > L
30 s chair stand	14.59 ± 5.19	15.32 ± 5.22	14.25 ± 5.32	<0.0001 ^a^	N > S > L
Back scratch	−9.65 ± 12.93	−9.20 ± 12.54	−11.36 ± 13.25	<0.0001 ^a^	N, S > L
Chair sit-and-reach test	2.57 ± 10.65	2.87 ± 10.42	0.93 ± 10.51	<0.0001 ^a^	N, S > L
8-foot up-and-go	8.36 ± 3.04	7.95 ± 2.85	8.74 ± 3.46	<0.0001 ^a^	L > S > N
One-leg stance with eye open	12.08 ± 10.03	13.22 ± 10.36	11.19 ± 9.98	<0.0001 ^a^	N > S > L
BMI (kg/m^2^)	24.73 ± 3.53	24.81 ± 3.42	24.81 ± 3.43	0.444	
WHR	0.89 ± 0.08	0.89 ± 0.08	0.90 ± 0.08	<0.0001 ^a^	L > N > S

Abbreviations: ANOVA, analysis of variance; BMI, body mass index; CEI, cardiorespiratory endurance index; S, short; L, long; N, normal; WHR, waist-to-hip ratio.; values are expressed as means ± standard deviation (SD) and %.; ^a^ one-way ANOVA, *p* < 0.05.

**Table 3:** Multivariate adjusted ORs for short sleep duration (≤5 h/day) in relation to each physical fitness measurement after adjustment for potential confounders.

Physical Fitness Levels	Factors Not Adjusted			Factors Adjusted ^a^		
	OR	95% CI	*p*	OR	95% CI	*p*
2 min step test						
Quartile 4	0.9868	0.8721–1.1165	0.8330	1.1422	0.9975–1.3078	0.0544
Quartile 3	0.9599	0.8548–1.0779	0.4890	1.0909	0.9606–1.2389	0.1800
Quartile 2	0.9587	0.8578–1.0716	0.4579	1.0302	0.9122–1.1635	0.6318
Quartile 1	1.0000	−	−	1.0000	−	−
30 s arm curl						
Quartile 4	0.9972	0.8868–1.1213	0.9625	0.9976	0.8756–1.1366	0.9713
Quartile 3	0.9895	0.8843–1.1072	0.8541	0.9651	0.8532–1.0917	0.5721
Quartile 2	1.0562	0.9508–1.1732	0.3083	1.0439	0.9307–1.1708	0.4630
Quartile 1	1.0000	−	−	1.0000	−	−
30 s chair stand						
Quartile 4	0.8730	0.7616–1.0006	0.0510	0.8948	0.7702–1.0396	0.1463
Quartile 3	0.9058	0.8049–1.0194	0.1006	0.9151	0.8045–1.0411	0.1776
Quartile 2	0.9972	0.892–1.1147	0.9604	1.0243	0.9070–1.1568	0.6983
Quartile 1	1.0000	−	−	1.0000	−	−
Back scratch						
Quartile 4	0.9901	0.8786–1.1158	0.8704	0.9415	0.8235–1.0765	0.3778
Quartile 3	1.1469	1.0241–1.2844	0.0177 ^b^	1.1084	0.9771–1.2573	0.1096
Quartile 2	1.0074	0.9031–1.1237	0.8952	0.9726	0.8622–1.0972	0.6516
Quartile 1	1.0000	−	−	1.0000	−	−
Chair sit-and-reach test						
Quartile 4	1.0489	0.9359–1.1756	0.4116	0.9987	0.8804–1.1328	0.9834
Quartile 3	1.0994	0.9841–1.2281	0.0936	1.0578	0.9356–1.1958	0.3698
Quartile 2	0.9757	0.8766–1.0861	0.6528	0.9591	0.8523–1.0793	0.4885
Quartile 1	1.0000	−	−	1.0000	−	−
8-foot up-and-go						
Quartile 4	1.4189	1.2367–1.6279	<0.0001 ^b^	1.0457	0.8953–1.2213	0.5725
Quartile 3	1.1808	1.0484–1.3298	0.0061 ^b^	0.9281	0.8131–1.0595	0.2695
Quartile 2	1.1013	0.9859–1.2302	0.0876	0.9582	0.8477–1.0831	0.4950
Quartile 1	1.0000	−	−	1.0000	−	−
One-leg stance with eye open						
Quartile 4	0.8695	0.7698–0.9821	0.0244 ^b^	0.9824	0.8549–1.1288	0.8019
Quartile 3	0.9373	0.8375–1.0490	0.2594	1.0223	0.9024–1.1581	0.7290
Quartile 2	0.9587	0.8596–1.0691	0.4483	0.989	0.8777–1.1144	0.8555
Quartile 1	1.0000	−	−	1.0000	−	−
BMI (kg/m^2^)						
Quartile 4	0.9045	0.8094–1.0107	0.0763	0.8660	0.7653–0.9800	0.0226 ^b^
Quartile 3	0.8334	0.7467–0.9303	0.0012 ^b^	0.8031	0.7119–0.9061	0.0004 ^b^
Quartile 2	0.9283	0.8360–1.0308	0.1639	0.9248	0.8253–1.0363	0.1784
Quartile 1	1.0000	−	−	1.0000	−	−
WHR						
Quartile 4	0.8758	0.7824–0.9803	0.0212 ^b^	0.9204	0.8100–1.0459	0.2033
Quartile 3	0.9058	0.8146–1.0071	0.0673	0.9187	0.8150–1.0356	0.1652
Quartile 2	0.9992	0.9006–1.1086	0.9881	1.0357	0.9247–1.1601	0.5441
Quartile 1	1.0000	−	−	1.0000	−	−

Abbreviations: BMI, body mass index; CEI, cardiorespiratory endurance index; CI, confidence interval; OR, odds ratio; WHR, waist-to-hip ratio.; ^a^ the logistic regression models adjusted for age, gender, education, monthly income, marital status, self-reported health status, smoking status, and chewing betel nuts; ^b^ logistic regression, *p* < 0.05.

**Table 4:** Multivariate adjusted ORs for long sleep duration (≥8 h/day) in relation to each physical fitness measurement after adjustment for potential confounders.

Physical Fitness Levels	Factors Not Adjusted			Factors Adjusted ^a^		
	OR	95% CI	*p*	OR	95% CI	*p*
2 min step test						
Quartile 4	0.9627	0.8712–1.0637	0.4550	0.9913	0.8903–1.1037	0.8733
Quartile 3	0.9387	0.8551–1.0306	0.1844	0.9737	0.8803–1.0769	0.6038
Quartile 2	1.0141	0.9281–1.1080	0.7573	1.0161	0.9237–1.1178	0.7425
Quartile 1	1.000	−	−	1.000	−	−
30 s arm curl						
Quartile 4	0.9517	0.8646–1.0477	0.3127	0.9597	0.8645–1.0655	0.4409
Quartile 3	1.0265	0.9379–1.1233	0.5705	1.0389	0.9429–1.1447	0.4401
Quartile 2	1.0553	0.9697–1.1483	0.2123	1.0900	0.9954–1.1935	0.0628
Quartile 1	1.000	−	−	1.000	−	−
30 s chair stand						
Quartile 4	0.8692	0.7786–0.9704	0.0126 ^b^	0.8367	0.7434–0.9417	0.0031 ^b^
Quartile 3	0.8733	0.7941–0.9604	0.0052 ^b^	0.8406	0.7593–0.9306	0.0008 ^b^
Quartile 2	0.8929	0.8161–0.9768	0.0134 ^b^	0.8722	0.7921–0.9604	0.0054 ^b^
Quartile 1	1.000	−	−	1.000	−	−
Back scratch						
Quartile 4	0.8357	0.7600–0.9189	0.0002 ^b^	0.8695	0.7831–0.9656	0.0089 ^b^
Quartile 3	0.927	0.8469–1.0146	0.0998	0.9381	0.8494–1.0360	0.2070
Quartile 2	0.9548	0.8778–1.0386	0.2812	0.9425	0.8603–1.0327	0.2042
Quartile 1	1.000	−	−	1.000	−	−
Chair sit-and-reach test						
Quartile 4	0.7609	0.6948–0.8333	<0.0001 ^b^	0.8069	0.7310–0.8907	<0.0001 ^b^
Quartile 3	0.8190	0.7502–0.8941	<0.0001 ^b^	0.8613	0.7828–0.9477	0.0022 ^b^
Quartile 2	0.8325	0.7668–0.9038	<0.0001 ^b^	0.859	0.7855–0.9395	0.0009 ^b^
Quartile 1	1.000	−	−	1.000	−	−
8-foot up-and-go						
Quartile 4	1.2922	1.1568–1.4435	<0.0001 ^b^	1.2708	1.1232–1.4378	0.0001 ^b^
Quartile 3	1.0587	0.9618–1.1654	0.2443	1.0246	0.9218–1.1388	0.6529
Quartile 2	1.0514	0.9614–1.1498	0.2724	1.0126	0.9181–1.1169	0.8021
Quartile 1	1.000	−	−	1.000	−	−
One-leg stance with eye open						
Quartile 4	0.7802	0.7074–0.8605	<0.0001 ^b^	0.8012	0.7178–0.8943	0.0001 ^b^
Quartile 3	0.8145	0.7441–0.8916	<0.0001 ^b^	0.8460	0.7662–0.9340	0.0009 ^b^
Quartile 2	0.8846	0.8111–0.9648	0.0056 ^b^	0.8744	0.7959–0.9605	0.0051 ^b^
Quartile 1	1.000	−	−	1.000	−	−
BMI (kg/m^2^)						
Quartile 4	0.8196	0.7486–0.8974	<0.0001 ^b^	0.8579	0.7768–0.9475	0.0025 ^b^
Quartile 3	0.9063	0.8300–0.9897	0.0285 ^b^	0.9058	0.8236–0.9963	0.0417 ^b^
Quartile 2	0.9284	0.8519–1.0118	0.0906	0.9161	0.8353–1.0047	0.0630
Quartile 1	1.000	−	−	1.000	−	−
WHR						
Quartile 4	1.2257	1.1202–1.3412	<0.0001 ^b^	1.1960	1.0812–1.3230	0.0005 ^b^
Quartile 3	1.2574	1.1545–1.3695	<0.0001 ^b^	1.2254	1.1144–1.3475	<0.0001 ^b^
Quartile 2	1.0683	0.9783–1.1665	0.1413	1.0503	0.9547–1.1553	0.3135
Quartile 1	1.000	−	−	1.000	−	−

Abbreviations: BMI, body mass index; CEI, cardiorespiratory endurance index; CI, confidence interval; OR, odds ratio; WHR, waist-to-hip ratio.; ^a^ the logistic regression models adjusted for age, gender, education, monthly income, marital status, self-reported health status, smoking status, and chewing betel nuts; ^b^ logistic regression, *p* < 0.05.

## Abbreviations

The following abbreviations are used in this manuscript:

ANOVA	analysis of variance
BMI	body mass index
CI	confidence interval
HC	hip circumference
HHPFST	National Physical Fitness Survey in Taiwan
OR	odds ratio
SD	standard deviation
SAS	statistical analysis system
SA	successful aging
SFT	serious fitness test
WC	waist circumference
WHR	waist-to-hip ratio
